# 

**Published:** 1858-05

**Authors:** 


					﻿PUBLISHED MONTHLY, AT S3 PER ANNUM IN ADVANCE.
z
©
3
o
g
©
w
c
©■
>»
©
&
©
c
*3
a
■
©
a
1
©
©
©
>*
fl
z
fl
©
Q
©
M
z
.©
S
©
©
©
©
>
©
©
fl
©
>•
a
x
c
©
©
H
z
S
©
©
©
©
©
a
l#
K
C
<
&-
rr
©
a
a' rr, a. x t . \
Mimi ffl 81IRUICAL
IOIif11•
EDITED BY
JOSEPH P. LOGAN, M. D.,
Professor of Physiology and General Pathology in the Atlanta Medical College.
AND
W. F. WESTMORELAND, M. D.,
Professor of the Principles and Practice of Surgery in the Atlanta Medical College,
Pax et scientia, sed veritas sine timore.
Vol. III.	MAY, 1858.	Ko. IX.
ATLANTA, GEORQffA^
PRINTED BY G- P- EDDY CO.
1 8 5 8.
EACH NUMBER CONTAINS SIXTY-FOUR PAGES.
9
' z
B
p
z
©
Z
fl
©
3
5
©
z
rt
©
a
©
©
©
5
•b
£
S
S
©
©
*
y
5.
1
B
ft
«
B
©
o
©
*
B-
©
B
S
©
©
d
W
>
r
5*
©
z
©
p
s
©
3'
©
l»
©
z
				

